# Screening Mammalian Cochlear Hair Cells to Identify Critical Processes in Aminoglycoside-Mediated Damage

**DOI:** 10.3389/fncel.2018.00179

**Published:** 2018-07-02

**Authors:** Hyun Woo Lim, Kwang Pak, Allen F. Ryan, Arwa Kurabi

**Affiliations:** ^1^Division of Otolaryngology, Department of Surgery, University of California, San Diego, La Jolla, CA, United States; ^2^Department of Otolaryngology, Gangneung Asan Hospital, University of Ulsan College of Medicine, Gangneung, South Korea; ^3^San Diego VA Healthcare System, La Jolla, CA, United States; ^4^Department of Neurosciences, School of Medicine, University of California, San Diego, La Jolla, CA, United States

**Keywords:** ototoxicity, antioxidants, kinase inhibitors, drug screen, hair cells

## Abstract

There is considerable interest in discovering drugs with the potential to protect inner ear hair cells (HCs) from damage. One means of discovery is to screen compound libraries. Excellent screening protocols have been developed employing cell lines derived from the cochlea and zebrafish larvae. However, these do not address the differentiated mammalian hair cell. We have developed a screening method employing micro-explants of the mammalian organ of Corti (oC) to identify compounds with the ability to influence aminoglycoside-induced HC loss. The assay is based on short segments of the neonatal mouse oC, containing ~80 HCs which selectively express green fluorescent protein (GFP). This allows the screening of hundreds of potential protectants in an assay that includes both inner and outer HCs. This review article describes various screening methods, including the micro-explant assay. In addition, two micro-explant screening studies in which antioxidant and kinase inhibitor libraries were evaluated are reviewed. The results from these screens are related to current models of HC damage and protection.

## Introduction

Ototoxicity is a significant side effect of some valuable medications that have been used for life-threatening diseases. Ototoxic drugs can cause irreversible damages in the inner ear that lead to loss of function in hearing and balance. Hearing loss is a serious handicap bearing impact on daily communication, quality of life, psychological status and employability. Moreover, vestibular damage increases the probability of falls, which are a leading cause of death or injury in the elderly. The clinical usefulness of potentially ototoxic drugs like aminoglycoside antibiotics and platinum-based anticancer drugs mandate their usage for very serious clinical conditions, in spite of their side effects. Aminoglycosides, including gentamicin, neomycin, and amikacin, play an important role in treating multi-drug resistance tuberculosis, neonatal sepsis, cystic fibrosis and pseudomonas-induced respiratory infections (Forge and Schacht, 2000). Unfortunately, aminoglycoside-induced ototoxicity occurs in as many as 50% of patients with multi-drug resistance tuberculosis and as many as 20% in children treated for cystic fibrosis (Al-Malky et al., 2015; Sagwa et al., 2015). Prevention of ototoxicity is thus a critical problem that has attracted considerable research attention to identify the mechanisms of inner ear damage and repair. Since other forms of sensorineural cell damage may occur via similar mechanisms, research on ototoxicity may also accelerate the development of treatments for other types of hearing loss.

Ototoxic drugs generally target the sensory hair cells (HCs) of the inner ear (Wong and Ryan, 2015). Aminoglycoside-induced HC death occurs initially in basal turn outer HCs, and then extends to more apical outer HCs and inner HCs with increasing cumulative dose (Ryan and Dallos, 1975; Forge and Schacht, 2000). The apices of supporting cells surrounding dying HCs expand, to seal the reticular lamina and maintain function in the surviving HCs (Leonova and Raphael, 1997; Steyger et al., 1997). Adjacent supporting cells and resident macrophages engulf the residual dead HCs (Monzack et al., 2015). Although HCs are the primary target of ototoxicity, the damaging effects may also extend to the afferent synapses of inner HCs and spiral ganglion neurons (Nicol et al., 1992; Koo et al., 2015; Oishi et al., 2015).

The cellular mechanisms that underlie HC stress and death are not fully understood. Nevertheless, mounting evidence indicates that ototoxins induce the formation and accumulation of free radicals like reactive oxygen species (ROS) and reactive nitrogen species (RNS) as an early step in the damage course (Yamane et al., 1995; Yamashita et al., 2004; Choung et al., 2009). Free radicals can cause damage to parts of cells such as proteins, DNA and cell membranes. The distortion of regulation of ROS and RNS signaling can trigger apoptotic enzymatic cascades and stimulate numerous intracellular signaling cascades including p38 mitogen-activated protein kinase (MAPK) and/or the Jun-kinase (JNK) signaling pathways (Eshraghi et al., 2007; Coffin et al., 2013). The activation of ROS and MAPK stress pathways may stimulate the sequential actions of caspases leading to cochlear HC apoptosis. (Yakovlev and Faden, 2001; Wang et al., 2003; Yang et al., 2004). Caspase-mediated cell death pathways have been widely implicated in the programmed cell death of HCs (Nicotera et al., 2003), although necrosis may also occur (Park et al., 2012).

A major motivation for the study of HC damage mechanisms is the search for means of prevention (O’Sullivan et al., 2017). However, given the complexity of intracellular signaling and other cellular processes, and the mixed results from clinical trials (e.g., Lindblad et al., 2011; Kopke et al., 2015), it seems likely that the as yet unidentified processes contribute to ototoxic HC damage. To better understand ototoxicity-related cellular processes and overcome the current knowledge limits regarding fundamental mechanisms, screening large numbers of compounds without *a priori* hypotheses of HC damage mechanisms has been employed.

In this review article, we discuss various screening methods for the assessment of large numbers of compounds to identify those that modify ototoxic damage to HCs. This includes assays based on inner ear cell lines and zebrafish larvae, as well as development of an assay based on organ of Corti (oC) micro-explants that allows the screening of differentiated mammalian cochlear HCs. We also summarize results from micro-explant screening and interpret those results linking them with reference to HC damage mechanisms.

## Ototoxicity Screening Assays

Obviously, the gold standard for preclinical evaluation of HC protectants is the *in vivo* animal model, which comes closest to the human situation and allows functional assessment using behavioral or electrophysiological measures. However, the difficulty and expense of evaluating compounds *in vivo*, coupled with the need for replication and dose-response assessment, severely limits the number of compounds that can be evaluated. For this reason, alternative assays have been developed.

### Inner Ear Cell Lines

A number of immortalized cell lines have been developed from the inner ear that express genes characteristic of HCs (e.g., Barald et al., 1997; Holley and Lawlor, 1997; Kalinec et al., 1999). Kalinec et al. (2003) demonstrated that one such line (HEI-OC1) was sensitive to ototoxins including aminoglycosides and cisplatin and could therefore be used to screen protective compounds by assessing caspase activity. This initial report was expanded upon (Kalinec et al., 2016) with extensive data on variability and a broader group of ototoxins. Inner ear cell lines have been used to evaluate many potential otoprotectants individually (e.g., Chang et al., 2001; Kim et al., 2008; Jadidian et al., 2015). However, few screening studies to identify HC protectants have been performed on inner ear cell lines. Recently, Teitz et al. (2016) developed an automated assay based on the HEI-OC1 line to screen 4385 unique bioactive compounds for the ability to protect against cisplatin-induced caspase activation. They obtained more than 150 hits that were appropriate for follow-up screening.

Cell lines have advantages that make them very attractive for a large-scale compound screen. Extremely large numbers of cells can be generated, permitting high-throughput screens of multiple compounds, doses and replicates. However, there are also disadvantages of cell lines as screening tools. These include the fact that the cell lines are not differentiated HCs. They express not only some HC genes but also some characteristic of supporting cells (Kalinec et al., 2003). In addition, these cell lines possess neither stereocilia or their mechano-electrical transduction (MET) channels, through which aminoglycosides can enter HCs.

### Zebrafish Lateral Line

Use of the larval zebrafish lateral line, in which HCs have been fluorescently labeled, to screen compounds for HC protective ability was initially developed by Ton and Parng (2005). They found that compounds that had been shown previously to protect HCs in mammals, glutathione (GSH), N-acetylcysteine (NAC), and d-methionine (d-dMET) also protected lateral line HCs in this model, indicating its utility for identifying protectants of mammalian HCs. Owens et al. (2008) subsequently adopted this model and screened a library of 10,960 compounds for the ability to protect lateral line HCs against high-dose neomycin. They identified two benzothiophene carboximides that were protective, leading to the development of a lead compound that protects mammalian HCs *in vivo* (Chowdhury et al., 2018). Ou et al. (2009) subsequently screened a library of 1040 FDA-approved drugs and bioactive compounds and identified seven that protected against neomycin damage. Two of these were shown to protect adult mouse utricular HCs *in vitro*. Vlasits et al. (2012) expanded upon this finding by evaluating the library for protection against multiple ototoxins. The screen revealed several compounds that were protective. Interestingly, the compounds were effective against different combinations of toxins, suggesting that multiple mechanisms of HC damage are involved, even across different aminoglycosides. To explore mechanisms of HC death, Coffin et al. (2013) used zebrafish to screen a custom library of cell death inhibitors. They found that a proteasome inhibitor protected lateral line HCs against gentamicin, neomycin and cisplatin. However, results from other inhibitors suggested the involvement of distinct cell death mechanisms for the different ototoxins. In a later study, Kruger et al. (2016) screened a library of 502 natural compounds for ability to protect against gentamicin or neomycin toxicity. They identified four bisbenzylisoquinoline derivatives that protected HCs by preventing aminoglycoside uptake into HCs. Kenyon et al. (2017) screened 160 ion-channel modulators for the ability to reduce gentamicin-induced HC death, block the HC MET channel or reduce gentamicin uptake. A total of 72 compounds affected one of these variables. Of these, 13 protected against HC damage in neonatal mouse oC explants, while six were transduction channel blockers. These studies illustrate the utility of the zebrafish model for identifying potential HC protectants. Recently, Philip et al. (2018) developed an automated HC damage-scoring algorithm based on machine-learning techniques that produces accurate damage assessment, which will enhance the speed and ease of use of this assay.

Advantages of the zebrafish assay include the ability to generate large numbers of larvae for high-throughout screening, and the fact that the HCs are *in vivo*. Also, the HCs are surrounded by supporting cells, which may modify responses to toxins or compounds, and are fully developed. This allows the determination of HC function through behavioral assays as well as MET channel function using channel-penetrating dyes such as FM1–43. Disadvantages of this model include the species difference between fish and mammals, with the potential for variation between the responses of lateral line HCs and those of the highly specialized outer and inner HCs of the oC.

### Inner Ear Sensory Epithelia

*In vitro* preparations of the neonatal rodent oC and neonatal or adult macular epithelium have been used to evaluate many compounds for their ability to protect against ototoxicity (e.g., Low et al., 1996; Corbacella et al., 2004; Lee et al., 2017). However, this assay is limited by animal numbers, and thus has been used primarily to assess compounds individually or in small numbers, rather than as a screening tool. It is interesting to note that hits from zebrafish and cell line screens are sometimes validated using rodent sensory epithelial cultures, prior to *in vivo* testing (e.g., Ou et al., 2009), since it provides access to differentiated mammalian HCs.

### Organ of Corti Micro-Explants

It would be advantageous to be able to screen larger numbers of compounds *in vitro* using differentiated mammalian HCs. To meet this need, we developed an *in vitro* assay using micro-explants of neonatal mouse oC (see Noack et al., 2017; Ryals et al., 2017; for details of methods). In brief, oC was harvested from the cochlea of postnatal day 3–5 *pou4f3*/eGFP (enhanced GFP) transgenic mouse pups, which selectively express eGFP in HCs (Masuda et al., 2011). Micro-explants of approximately 20 inner HCs and 60 associated outer HCs from the basal and middle turns were exposed to 200 μM gentamicin plus a candidate compound (at the high gentamicin dose used, we saw no difference in HC damage between the basal and middle turns). HCs were imaged before and 1, 2 and 3 days after the application of gentamicin to evaluate HC survival. A “hit” was defined as any compound that had HC counts deviating significantly from controls. Hits were validated by retesting.

This assay has the advantage that the responses of mammalian inner and outer HCs are evaluated. It can be argued that the mammalian oC, even in the developing state, provides results that are more applicable to humans than other *in vitro* assays. In addition, micro-explants allow for a greater number of compounds to be evaluated simultaneously than would be practical to screen with whole sensory epithelial explants or *in vivo*. Although the assay is by no means high-throughput, it will readily allow the evaluation of a few hundred compounds. In addition, HC-specific fluorescent GFP expression allows continuous monitoring of HC survival throughout the period of the screening assay, instead of a snapshot provided by HCs that must be labeled at the end of an experiment. This eliminates the need for multiple samples to evaluate different times of treatment, as well as HC staining. Finally, because the assay is normalized to relative uniformity, it not only allows hit identification, but also assesses the relative effectiveness between different compounds for toxicity and protection.

This micro-explant assay also has limitations. As noted above for whole epithelia, the assay is based on young neonatal HCs which are not functionally mature yet. Also, the number of compounds and conditions that can be tested is limited in comparison to high-throughput cell-lines or zebrafish larvae assays. Finally, like other screening methods, candidates found through the micro-explant screening assay need to be confirmed by postscreen characterization of hits and testing in *in vivo* mammals. These limitations must be considered when interpreting the results from micro-explants assay studies. The oC micro-explant assay was used to screen a redox compound library (Noack et al., 2017) and a kinase inhibitor library (Ryals et al., 2017).

### Redox Library Screen

**Figure 1:**
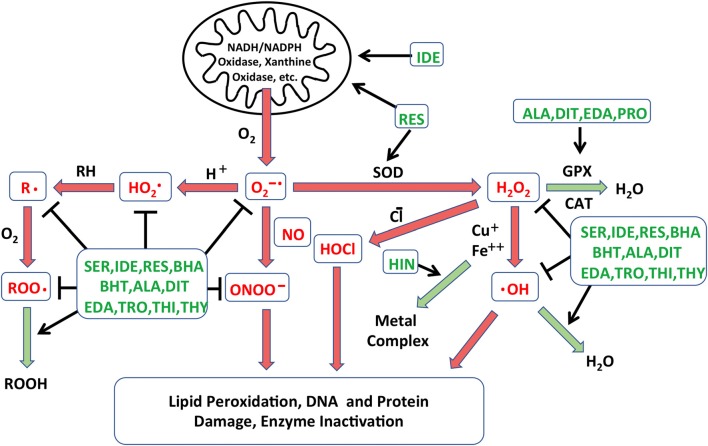
Schematic diagram illustrating the different processes leading to free radical formation. The creation of reactive oxygen species (ROS) in cells is combated by the action of native antioxidants enzyme systems that catalyze these reactions. Reactions that are damaging to cells are depicted in red arrows, while those leading to free radical neutralization are in green arrows. Points at which the antioxidants identified in the Noack et al. (2017) screen may intercede are indicated in green text. NADH: nicotinamide adenine dinucleotide; NADPH: NADH phosphate; O_2_^−•^: superoxide anion; HO_2_^•^: perhydroxyl radical; •OH: hydroxyl radical; H_2_O_2_: hydrogen peroxide; HOCl: hypochlorous acid; ONOO^−^: peroxynitrite; R•: lipid alkyl radical; RH: lipid, ROO•: lipid peroxyl radical; ROOH: lipid hydroperoxide; GPX: glutathione peroxidase; CAT: catalase; SOD: superoxide dismutase; SER: seratrodast; IDE: idebenone; RES: resverastrol; ALA: DL-alpha-lipoic acid; HIN: hinokitiol; EDA: edaravone; PRO: procysteine; TRO: trolox; THI: thiourea; THY: thymoquinone; BHA: butylated hydroxyanisole; BHT: butylated hydroxytoluene; DIT: dithitreitol.

The generation and resolution of free radicals are illustrated schematically in Figure 1. Production of excess radical species has been widely accepted as an early event of HC damage following ototoxic or noise induced inner ear damage (Yamane et al., 1995; Yamashita et al., 2004; Huth et al., 2011). The accumulation of free radicals is detected within HCs well before any morphological signs of damage are documented, suggesting their roles in initiation of the ototoxic process (Choung et al., 2009). Free radicals cause cellular damage by reacting with DNA, cytosolic molecules, cellular receptors, and membrane lipids. This leads to the activation of cell stress pathways (see below), which is followed by cellular loss through cell breakdown and/or apoptosis (Martindale and Holbrook, 2002). ROS can also increase production of inflammatory mediators, such as tumor necrosis factor-α (TNF-α ) and interleukin-6 (IL-6), which can induce cochlear damage (Keithley et al., 2008; Wakabayashi et al., 2010; Tan et al., 2016). Mitochondria are the major source of free radicals, generating reactive species as a byproduct of metabolism. Aminoglycosides can disrupt calcium homeostasis between endoplasmic reticulum (ER) and mitochondria in tissues including the inner ear. In other systems, enhanced flow of Ca^2+^ from ER to mitochondria induces increased mitochondrial membrane permeability and release of ROS into cytoplasm (Batandier et al., 2004).

The overproduction of ROS in cells is combated by the action of native antioxidant enzyme systems. GSH, superoxide dismutase (SOD), GSH reductase, GSH peroxidase, catalases and coenzyme Q11 have all been detected in cochlear tissues (Jacono et al., 1998; McFadden et al., 1999; Fetoni et al., 2013). Systemic or locally applied antioxidants have also been studied as prophylaxis to enhance cochlear defense against ototoxin-induced ROS. Treatments with antioxidants have shown protective effects in ROS-induced HC loss in animal studies (Garetz et al., 1994; Rybak et al., 1999; Sha et al., 2001), while the results of clinical trials are more mixed (Kramer et al., 2006; Lindblad et al., 2011; Bagger-Sjöbäck et al., 2015).

Antioxidants vary considerably in their mechanisms of action. Their possible actions include radical scavenging of species that initiate peroxidation, singlet oxygen quenching ability, chelating redox metals, breaking free radical chain reactions, reducing the concentration of O_2_, and/or stimulating endogenous antioxidant enzymes (Lu et al., 2010). Due to the different mechanism(s) of action and the variations in effectiveness of individual antioxidant compounds, we felt that screening a library of antioxidants would potentially be productive in identifying novel antioxidants, as well as in comparing the relative efficiency of different antioxidants and antioxidant classes.

A library of 81 antioxidants was screened using the developed *in vitro* explant assay. The library contained several compounds that have been previously studied as potential treatments for HC damage, together with many more antioxidants that have never been applied to ototoxicity. Thirteen antioxidants exhibited significant protection of HCs, while six proved to be damaging. Of the protective antioxidants from the screening, eight antioxidants had previously been shown to be protective for HCs against oxidative stress: idebenone, resverastrol, DL-alpha-lipoic acid, MC-186, procysteine, trolox, thiourea and thymoquinone, while five antioxidants, seratrodast, butylated hydroxyanisole, hinokitiol, butylated hydroxytoluene and dithitreitol, have not been shown to reduce oxidative HC damage. The recognized modes of action of all of the protective antioxidants are shown in Figure 1. It is clear from the figure that no one mode of action determined the ability of antioxidants to protect HCs. This suggests that gentamicin damages HCs by a number of redox mechanisms. Similarly, in the protective compounds, several different classes of antioxidants were present. While the antioxidants belonging to the quinone class, seratrodast and idebenone, were the most potent antioxidants, several other categories were also effective, and several other quinone antioxidants in the library failed to provide protection. These results demonstrate the value of screening without *a priori* knowledge of mechanism or compound class.

Perhaps the most surprising result was the lack of protection by most antioxidants, including a number publicized to preserve hearing and protect HCs in previous studies (e.g., Garetz et al., 1994; Holley and Nishida, 1995; Bas et al., 2012). This could be interrelated to the relatively very high dose of gentamicin used (200 μM). This concentration was chosen because it showed significantly reduced HC survival on day 1 and through day 3 in culture.

### Kinase Inhibitor Screen

Phosphorylation is a prevalent means of post-translational modification of proteins. It plays an important role in intracellular signaling and regulating intracellular processes, including both cell damage and survival (Hunter, 1995). Intracellular signaling pathways link the various cellular processes to the cell nucleus, activating gene expression programs that can be influential determinants of cell fate. Many damage and survival pathways can be activated by exposure to ROS (Martindale and Holbrook, 2002). Therefore, we screened a library of 160 kinase inhibitors targeting specifically all the major families in the kinome, using the oC micro-explant assay (Ryals et al., 2017). Of those, 15 exhibited a significant protective effect: epidermal growth factor receptor (EGFR) Inhibitors I and PP3, PDGF Inhibitors I and IV, Compound C, p38 Inhibitors I and III, AKT inhibitors V and X, Cdk4 Inhibitor II, Fascaplysin, Flt-3 inhibitor, Compound C, Casein Kinase 2 Inhibitor III and protein kinase R (PKR) Inhibitor. As in the antioxidant screen, some of the targets of the kinase inhibitors have been associated preciously in HC loss, while others are new.

**Figure 2:**
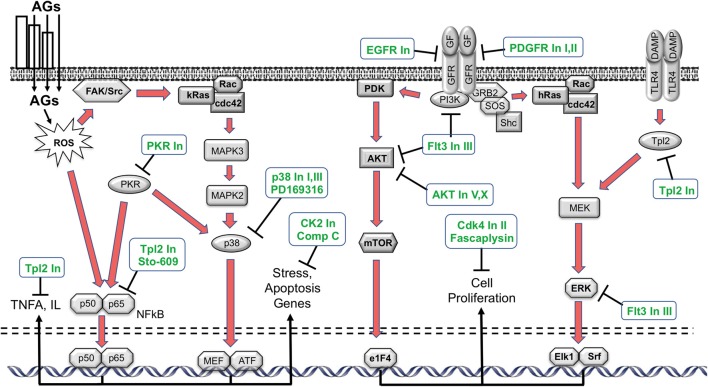
Schematic diagram illustrating the various intracellular damage signaling cascades (red arrows) implicated by the kinase inhibitor screen (Ryals et al., 2017), and the points at which the inhibitors identified in the screen (green text) are predicted to intercede. Of the 160 inhibitors, 15 exhibited a statistically significant protective effect (ANOVA). The inhibitors appear to target different molecules and point to potential kinase pathway contributions to ototoxicity. AGs: aminoglycosides; FAK: focal adhesion kinase; Rac: Ras-related C3 botulinum toxin substrate; cdc42: cell division control protein 42 homolog; MEF: myocyte enhancer factor; ATF: Activating transcription factor; PDK: Phosphoinositide-dependent kinase; AKT: protein kinase B; mTOR: mechanistic target of rapamycin; e1F4: eukaryotic initiation factor 4; GF: growth factor; GFR: GF receptor; PI3K: phosphoinositide 3-kinase; GRB2: growth factor receptor-bound protein 2; SOS: son of sevenless; MEK: MAPK/ERK kinase; ERK: extracellular receptor kinase; Elk1: ETS domain-containing protein; Srf: serum response factor; Tpl2: tumor progression locus 2; DAMP: damage-associated molecular pattern; TLR4: Toll-like receptor.

HC damage processes implicated by the kinase inhibitor screen are illustrated schematically in Figure 2. The screen suggests that p38/JNK signaling is involved. These stress pathways have been shown in many previous studies to play a role in aminoglycoside ototoxicity (e.g., Wei et al., 2005; Bas et al., 2012). Similarly, inhibitors of nuclear factor kappa β (NFκβ) signaling have been found to reduce HC damage due to ototoxin exposure in earlier studies (Nagy et al., 2005). The protective effects of EGFR inhibition have not been noted before, although EGFR inhibitors have exhibited a protective effect from ROS-mediated damage in other tissues (Liang et al., 2015). It is possible that EGF induces damaged HCs to attempt to enter the cell cycle, which would be fatal for these highly differentiated cells. A similar mechanism could mediate the responses to the platelet derived growth factor receptor (PDGFR) inhibitors identified as protective. These inhibitors also have other targets, including cSrc and cKit, which could mediate their action. PKR is the central constituent of the metainflammasome, which induces inflammation in response to ROS and ER stress (Kroemer et al., 2010). ER stress has been implicated in aminoglycoside toxicity to HCs (Oishi et al., 2015), although the role of PKR has not been previously noted. Compound C inhibits adenosine monophosphate-activated protein kinase (AMPK) and AMPK signaling can lead to apoptosis via e2F1. Noise-induced HC loss has been shown to be reduced by AMPK inhibition (Hill et al., 2016). Casein Kinase 2 can also mediate apoptosis, which may explain the effects of Casein Kinase Inhibitor III, which is another novel protectant.

AKT activation has previously been shown to enhance cell survival (Brand et al., 2011), thus our finding that AKT Inhibitors V and X enhance cell survival is in opposition to prior findings. However, under the right circumstances, AKT can induce cells to enter the cell cycle via p27^Kip1^ phosphorylation (Zhu et al., 2012). As noted above, differentiated mammalian HCs cannot survive cell cycle entry. It may be that the high dose of gentamicin used in the present study induces attempted cell cycle entry. This could explain the observed effects of AKT inhibition. Similarly, while extracellular receptor kinase (ERK) activation has also been found to be protective of HCs (Battaglia et al., 2003), it can also induce cell proliferation (Seger and Krebs, 1995). Thus, TLP2 Inhibitor or Flt3 Inhibitor III may protect HCs by inhibiting ERK. Ft3 inhibition can also reduce NFκβ activation, which could also contribute to its effect. Reducing forced cell cycle entry may also explain the protective effects of fascaplysin, and Cdk4 inhibitor and Cdk4 Inhibitor II.

## Conclusions

Screening assays have contributed significantly, both to the search for compounds that can protect HCs from damage, as well as to our understanding of HC damage mechanisms. The primary advantage of screening is that no *a priori* hypothesis regarding damage or protective mechanisms is involved. This permits the discovery of entirely novel protectants, and the identification of unsuspected damage and protective mechanisms. It also permits the comparison of large numbers of compounds that may address the same or similar cellular processes, to identify those that are most effective.

The micro-explant assay described here represents a new means of evaluating larger, numbers of compounds for potential leads and molecules that can be further optimized into drug candidates. This has been previously possible only using mammalian inner and outer HC lines. It is possible to screen hundreds of compounds for protective effects using this technique. The assay could also be applied to validate compound hits from high-throughput systems such as cell lines or zebrafish larvae, before moving to *in vivo* evaluation. The assay can also provide evidence of relative effectiveness for mammalian HCs.

As noted above, it is obvious that compounds identified with this screening assay need to be validated in adult HCs *in vivo*, a process that is underway for the top compounds identified in the two screens reviewed here.

Both screening assays suggest that the HC damage induced by aminoglycosides occurs by diverse mechanisms. The results provide evidence supporting a number of free radical damage mechanisms, including oxidative damage to various cellular constituents, Fenton and Fenton-like reactions involving redox metals, and opposing mechanisms of antioxidant enzyme systems and enhanced mitochondrial integrity and function. Similarly, a variety of cellular kinase processes and pathways appear to contribute to HC damage.

When comparing antioxidants vs. kinase inhibitors, it should be noted that the most effective antioxidants were able to provide more complete HC protection than the most effective kinase inhibitors. This may be because oxidative damage occurs early in aminoglycoside ototoxicity, well before HCs show any other signs of damage (Choung et al., 2009). In addition, as shown in Figure 1, antioxidants can influence oxidative damage at several points in the process. In contrast, cell signaling occurs later in the damage process. Moreover, many parallel processes and pathways appear to contribute to HC loss, and inhibitors target only one of a few components of the damage mechanisms.

In summary, the complexity of HC damage processes supports the use of compound screening. The fact that *a priori* reasoning would not have identified many of the compounds found to be effective or to lack protective ability also indicates that testing a large number of compounds can be effective in identifying new avenues for potential therapy to prevent aminoglycoside ototoxicity.

## Author Contributions

HL preformed the literature review and wrote the original manuscript text. AK, KP and AR contributed to manuscript writing. KP did the *in vitro* assay assessment. AR conceived the figures. All authors reviewed and approved the manuscript.

## Conflict of Interest Statement

AR is a cofounder of Otonomy Inc., serves as a consultant and member of the Scientific Advisory Board, and holds an equity position in the company. This relationship has been approved by the UCSD and San Diego VA Committees on Conflict of Interest. Otonomy Inc. played no part in the research reported here. The remaining authors declare that the research was conducted in the absence of any commercial or financial relationships that could be construed as a potential conflict of interest.

## Abbreviations

AMPK: adenosine monophosphate-activated protein kinase
eGFP: enhanced green fluorescent protein
EGFR: epidermal growth factor receptor
ER: endoplasmic reticulum
ERK: Extracellular receptor kinase
GFP: green fluorescent protein
HCs: hair cells
IL-6: interleukin-6
JNK: Jun-kinase
MAPK: mitogen-activated protein kinase
NFκβ: nuclear factor kappa β
oC: organ of Corti
PDGFR: platelet derived growth factor receptor
PKR: protein kinase R
RNS: reactive nitrogen species
ROS: reactive oxygen species
SOD: superoxide dismutase
TNFα: tumor necrosis factor-α.
